# The Influence of Sesquiterpenes from *Myrica rubra* on the Antiproliferative and Pro-Oxidative Effects of Doxorubicin and Its Accumulation in Cancer Cells

**DOI:** 10.3390/molecules200815343

**Published:** 2015-08-21

**Authors:** Martin Ambrož, Iva Boušová, Adam Skarka, Veronika Hanušová, Věra Králová, Petra Matoušková, Barbora Szotáková, Lenka Skálová

**Affiliations:** 1 Department of Biochemical Sciences, Faculty of Pharmacy, Charles University in Prague, Heyrovského 1203, Hradec Králové CZ-500 05, Czech Republic; E-Mails: ambrozm@faf.cuni.cz (M.A.); iva.bousova@faf.cuni.cz (I.B.); adam.skarka@outlook.com (A.S.); matousp7@faf.cuni.cz (P.M.); barbora.szotakova@faf.cuni.cz (B.S.); 2 Department of Medical Biology and Genetics, Faculty of Medicine, Charles University in Prague, Šimkova 870, Hradec Králové CZ-500 38, Czech Republic; E-Mails: hanusovaV@lfhk.cuni.cz (V.H.); kralovaV@lfhk.cuni.cz (V.K.)

**Keywords:** terpenes, cytotoxicity, *Myrica rubra*, antiproliferative effect, drugs combinations

## Abstract

The sesquiterpenes β-caryophyllene, β-caryophyllene oxide (CAO), α-humulene (HUM), *trans*-nerolidol (NER), and valencene (VAL) are substantial components of the essential oil from *Myrica rubra* leaves which has exhibited significant antiproliferative effects in several intestinal cancer cell lines, with CaCo-2 cells being the most sensitive. The present study was designed to evaluate the effects of these sesquiterpenes on the efficacy and toxicity of the anticancer drug doxorubicin (DOX) in CaCo-2 cancer cells and in primary culture of rat hepatocytes. Our results showed that HUM, NER, VAL and CAO inhibited proliferation of CaCo-2 cancer cells but they did not affect the viability of hepatocytes. CAO, NER and VAL synergistically potentiated the efficacy of DOX in cancer cells killing. All sesquiterpenes exhibited the ability to selectively increase DOX accumulation in cancer cells and did not affect DOX concentration in hepatocytes. Additionally, CAO and VAL were able to increase the pro-oxidative effect of DOX in CaCo-2 cells. Moreover, CAO mildly ameliorated DOX toxicity in hepatocytes. Based on all results, CAO seems to be the most promising compound for further testing.

## 1. Introduction

Sesquiterpenes, secondary metabolites produced mainly in higher plants, but also in fungi and invertebrates, possess promising anti-inflammatory, anti-parasitic and anti-carcinogenic activities [1,2,3]. The mechanism of the anti-cancer activity of sesquiterpenes is mostly mediated through the induction of apoptosis, intervention in the cell cycle, as well as the inhibition of proangiogenic, invasive and proliferative factors [4,5,6].

Besides their own anticancer effects, several sesquiterpenes were also able to improve the anti-cancer efficacy of common cytostatics when used in combinations. For example, β-caryophyllene potentiated the anti-cancer activity of paclitaxel in MCF-7, DLD-1 and L-929 cell lines as well as increased the intracellular accumulation of paclitaxel-Oregon green, probably due to the alteration of membrane permeability [7]. In addition, β-caryophyllene oxide enhanced the cytotoxic and pro-apoptotic effects of the chemotherapeutics paclitaxel and doxorubicin in human multiple myeloma and human prostate cancer cells [8]. Zerumbone, another sesquiterpene, enhanced the cytotoxic effects of cisplatin [9], while furanodiene increased the efficacy of tamoxifen and paclitaxel [10,11].

Sesquiterpenes represent the main components of plant essential oils [12] and this is likely the reason several essential oils exhibit positive activity against different cancer cell lines [13,14,15]. In a previous project of ours, the antiproliferative effects of the essential oil from *Myrica rubra* (Lour.) Sieb. et Zucc. (*Myricaceae*) were studied. [16] *Myrica rubra* is a subtropical Asian fruit tree that has been used in Chinese, Japanese and Taiwanese traditional medicine for more than 2000 years [17]. *M. rubra* essential oil proved to be non-toxic for non-cancerous cells and it significantly inhibited proliferation of several intestinal cancer cell lines, with CaCo-2 being the most sensitive [16]. These promising results instigated further testing of the antiproliferative effects of the main components of *M. rubra* essential oil and the evaluation of their potential use in combination with common cytostatics.

The anthracycline antibiotic doxorubicin (DOX) ranks among the most important cytostatics used in cancer therapy, but unfortunately its anti-cancer effect is frequently insufficient and severe toxicities occur in healthy tissues. Therefore, a search for possibilities of ways to increase DOX efficacy in cancer cells and minimize associated toxicities to non-cancerous tissues remains at the vanguard of scientific research [18]. Combinations of DOX with a number of natural compounds represent one possible approach.

**Figure 1:**
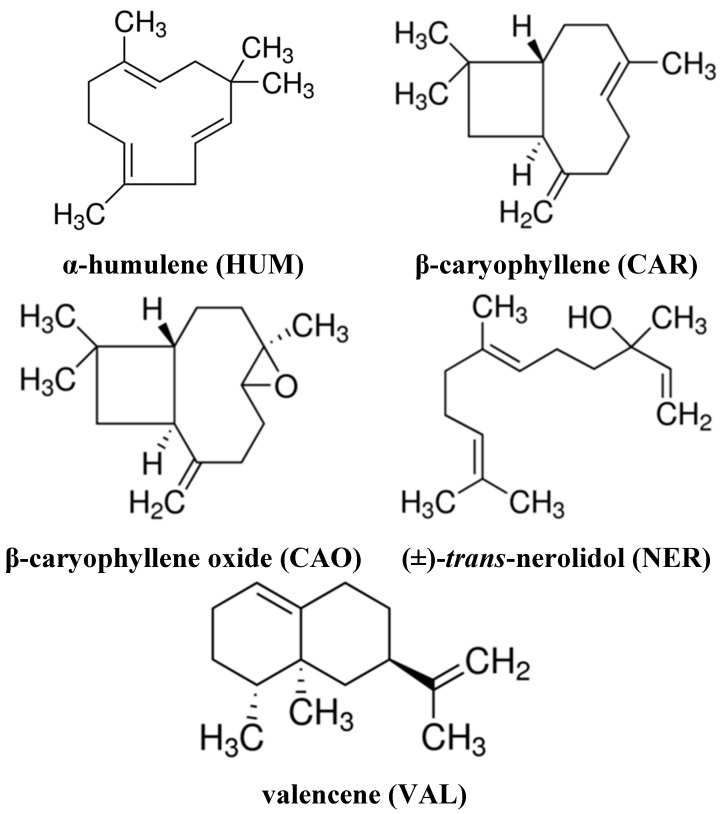
Structures of the tested sesquiterpenes.

The present study was designed to evaluate the effects on the efficacy of DOX in cancer cells and non-cancerous cells of selected sesquiterpenes (β-caryophyllene, CAR; β-caryophyllene oxide, CAO; α-humulene, HUM; (±)**-***trans*-nerolidol, NER; valencene, VAL; structures are presented in Figure 1) which are substantial components of *M. rubra* essential oil.

For this purpose, colon adenocarcinoma cells were used since these types of cancer are especially suitable for *peroral* treatment with natural products. The cell line CaCo-2 was chosen as this line showed the highest susceptibility to *M. rubra* essential oil in our previous study [16]. A primary culture of rat hepatocytes served as a model of non-cancerous cells. With the goal of determining the mechanism of the action of sesquiterpenes, their effects on DOX-mediated oxidative stress and the accumulation of DOX in cells was also studied.

## 2. Results and Discussion

An increasing resistance of mammalian tumor cells to chemotherapy along with the severe side effects of conservative medication have potentiated the search for new, alternative anticancer agents from natural sources. The widespread use of Chinese bayberry (*Myrica rubra*) in traditional medicine motivated the study of its biological activities, including anticancer efficacy. Also the identification of the active compounds of *M. rubra* that are responsible for its biological activities has become an important task [19]. In our previous study we tested the antiproliferative effect of *M. rubra* essential oil (MEO) in human colon and ileocecal adenocarcinoma cell lines HCT8, SW620, SW480, HT29 and CaCo-2. MEO significantly inhibited cell proliferation in a concentration-dependent manner in all cell lines, with CaCo-2 being the most sensitive. In cancer cells, MEO induced apoptosis but it did not affect the viability of isolated hepatocytes (as a model of normal non-cancerous cells) [16]. These promising results raised the question of what components could be primarily responsible for the anticancer effects of MEO. GC × GC-TOFMS analysis of the chemical composition of MEO revealed β-caryophyllene (43%), α-humulene (22%), humulene epoxide I (8%), valencene (6%), epi-α-selinene (6%), γ-muurolene (4%), β-caryphyllene oxide (3%), and *trans*-nerolidol (2%) as dominant compounds [16]. Of these sesquiterpenes, β-caryophyllene (CAR), α-humulene (HUM), valencene (VAL), β-caryphyllene oxide (CAO), and *trans*-nerolidol (NER) were selected for testing, as they were commercially available and their anticancer activities have previously been reported [3].

### 2.1. Effect of Individual Sesquiterpenes on Cell Proliferation/Viability

Firstly, the antiproliferative effect of five sesquiterpenes CAR, CAO, HUM, NER, VAL was tested in CaCo-2 cancer cells. The cells were incubated with individual sesquiterpenes in concentrations 0–50 µg/mL for 72 h. After incubation, a number of viable cells was assayed using a NRU test and IC_50_ values were calculated. 

**Figure 2:**
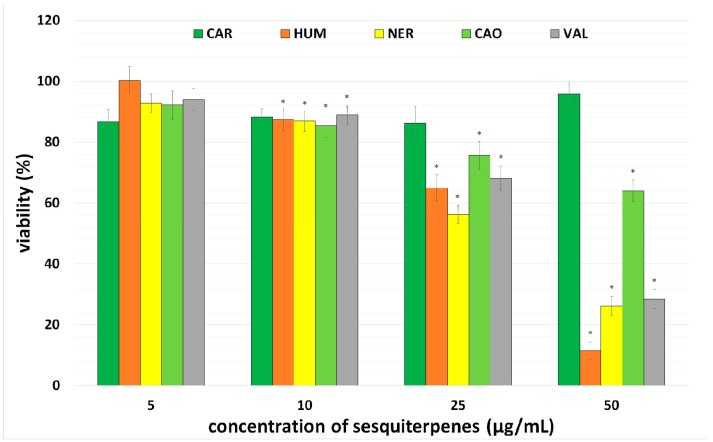
Effect of sesquiterpenes on the proliferation of cancer cell line CaCo-2. Viability was assayed using a NRU test. Data presented as percentage of control cells (=100%) represent the mean ± S.D. from 3 independent experiments (with 6 parallels in each). The asterisk indicates a significant difference from the control cells (*p* < 0.05).

All sesquiterpenes (with the exception of CAR) inhibited cancer cell proliferation in a concentration-dependent manner, with HUM and NER being the most effective. Based on this finding, CAR was excluded from further testing. Results are presented in Figure 2 and Table 1. Our results correspond with previously published studies in which HUM exhibited a marked antiproliferative effect in cancer cells, whereas CAR was found inactive [20,21]. In lung carcinoma cells A-549 and colon adenocarcinoma cells DLD-1, antiproliferative activity of HUM and NER was observed [22], while VAL exhibited an antiproliferative effect in Hela cells [23]. CAO evidenced potent cytotoxic activity against HepG2, AGS, HeLa, SNU-1, and SNU-16 cells in both a dose-dependent and time-dependent manner [24]. Significant suppression of cell proliferation and induction of apoptosis by CAO in human prostate and breast cancer cells was also reported [25]. CAO also potentiated TNF α-induced apoptosis and inhibited cancer cell invasion [8].

**Table 1:** IC_50_ values of individual sesquiterpenes in CaCo-2 cells after 72 h incubation.

	IC_50_ (µg/mL)
CAR	-
HUM	24.4 ± 2.4
VAL	38.1 ± 2.4
CAO	57.7 ± 3.9
NER	28.7 ± 2.5

With respect to the content of the tested sesquiterpenes (2%–22%) in MEO and comparing IC_50_ values of MEO (30 µg/mL) and individual sesquiterpenes (24–58 µg/mL), it is clear that none of the tested sesquiterpenes can be independently responsible for the MEO antiproliferative effect in CaCo-2 cells. This indicates that MEO possesses stronger activity probably due to the synergistic effect of HUM with other compounds identified in MEO. Legault and Pichette described a significant enhancement of HUM anticancer activity against MCF-7 cells by co-application with CAR, in contrast to the application of HUM alone [7].

**Figure 3:**
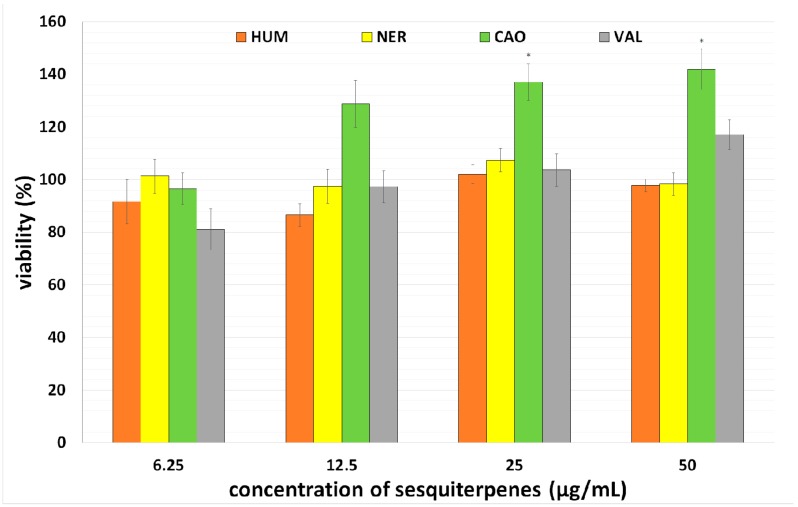
Effect of sesquiterpenes on viability of primary culture of rat hepatocytes assayed using a MTT test. Data presented as percentage of control cells (=100%) represent the mean ± S.D. from 2 independent experiments (with 6 parallels in each). The asterisk indicates a significant difference from the control cells (*p* < 0.05).

Consequently, the toxicity of selected sesquiterpenes was tested in primary culture of rat hepatocytes. When hepatocytes were incubated with CAO, HUM, NER and VAL in concentrations 0–50 µg/mL for 24 h, none of tested sesquiterpenes showed hepatotoxicity (see Figure 3). Actually, CAO mildly increased the viability of hepatocytes in a concentration-dependent manner. This positive finding is strengthened by the fact that CAO was not genotoxic in normal cells [26] and showed neuroprotective effects [27].

### 2.2. Effect of Sesquiterpenes in Combinations with Doxorubicin

The second part of present project was focused on sesquiterpenes in combination with the cytostatic drug doxorubicin (DOX). Combining drugs to increase their therapeutic effects, to reduce toxicity and to minimize drug resistance is becoming increasingly important in the treatment of cancer and can offer a favorable therapeutic outcome. Sesquiterpenes seem to be interesting candidates in combination therapy due to their possible penetration enhancing [28,29,30] and antioxidant/pro-oxidant effects [3]. DOX, a classic anticancer drug, is a mainstay of cancer chemotherapy, but clinical limitations arise from its cardiotoxicity and high incidence of multi-drug resistance. The use of DOX in combination represents one possible way of overcoming these limitations and improving DOX efficacy in cancer therapy, thus we decided to test the effect of these sesquiterpenes on the toxicity of DOX in hepatocytes and CaCo-2 cells.

**Figure 4:**
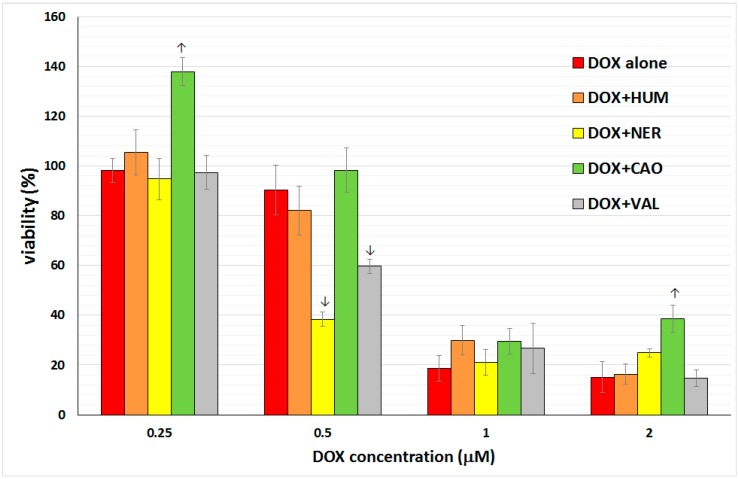
Effect of DOX (0.25, 0.5, 1, 2 µM) and the combinations 0.25 µM DOX + 6.25 µg/mL sesquiterpene, 0.5 µM DOX + 12.5 µg/mL sesquiterpene, 1 µM DOX + 25 µg/mL sesquiterpene, 2 µM DOX + 50 µg/mL sesquiterpene on viability of primary culture of rat hepatocytes assayed using a MTT test. Data presented as percentage of control cells (=100%) represent the mean ± S.D. from 2 independent experiments with 6 parallels in each (↑ significant increase; ↓ significant decrease).

The toxicity of DOX alone as well as combinations of DOX and sesquiterpene was tested in primary culture of rat hepatocytes. Hepatocytes were incubated with DOX alone (concentration range 0.25, 0.5, 1, 2 µM), with individual sesquiterpenes alone (concentrations 6.125, 12.5, 25, 50 µg/mL) and in combinations of DOX + each sesquiterpene. The number of viable cells was measured after 24 h exposition of cells to various concentrations of the tested compounds. The results (see Figure 4) showed a marked hepatotoxicity of DOX. HUM did not affect DOX toxicity. VAL and NER increased DOX toxicity at 0.5 µM concentration, but did not influence DOX toxicity at other concentrations. Conversely, CAO was able to significantly ameliorate DOX toxicity at concentrations 0.25 and 2 µM.

CaCo-2 cancer cells were incubated with DOX alone (concentrations 0.25, 0.5, 1, 2, 4 µM), with individual sesquiterpenes alone (concentrations 6.125, 12.5, 25, 50 µg/mL) and with DOX (concentrations 0.25, 0.5, 1, 2 µM) in combinations with each sesquiterpene (concentrations 6.125, 12.5, 25, 50 µg/mL). Concentrations of DOX and sesquiterpenes were selected according to the Chou-Talalay Method [31]. After 72-h incubation, the number of viable cells was assayed using NRU. Results showed a significant antiproliferative effect of DOX (IC_50_ 1.9 µM). When DOX was used in combinations with individual sesquiterpenes, a significant increase of DOX efficacy was observed.

**Figure 5:**
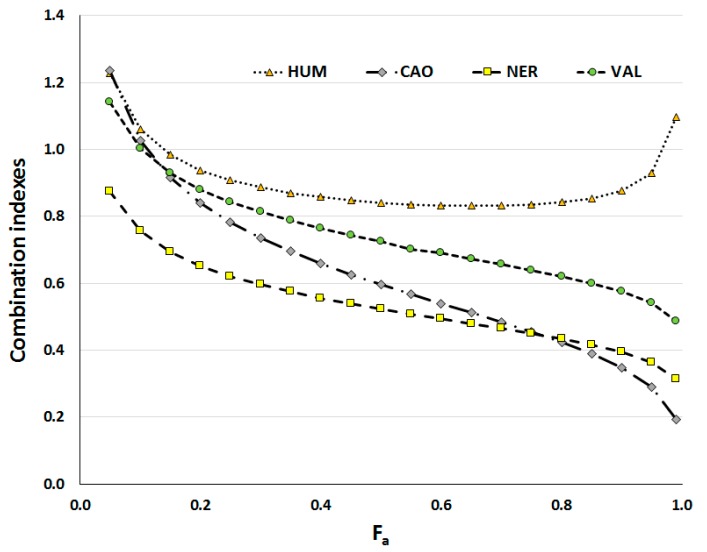
Combination indexes (CI) of DOX and sesquiterpenes in dependence on fraction of affected CaCo-2 cells (F_a_). Incubations lasted for 72 h. Data were calculated using CalcuSyn software.

With the aim of evaluating and quantifying the combined effect of DOX and sesquiterpenes, combination indexes were calculated using CalcuSyn (ver. 1.1, Biosoft, Cambridge, UK); the plot of combination indexes (CI) *vs.* fractions of dead cells (F_a_) is presented in Figure 5. DOX with HUM acted rather additively (especially in higher concentrations), but VAL, CAO and NER had significant synergistic effects with DOX, with this synergism increasing with higher concentrations (higher number of affected cells). Among sesquiterpenes, NER had the most synergistic effect on DOX at lower F_a_, while CAO effect was the strongest when a higher number of cells was affected (F_a_ > 0.8). It is important to emphasize that synergy of anticancer agents at high effect levels (F_a_ > 0.8) is more relevant to therapy than at low levels [31].

The synergism of DOX with CAO, VAL and NER was much stronger than the synergistic effect of DOX with MEO (Ambrož *et al.*—Submitted) although sesquiterpenes alone had a lower antiproliferative effect than MEO. In other studies, the antitumor activity of DOX was enhanced as a function of time when *Nigella sativa* oil (which contains sesquiterpenes as main components) was involved as an adjunct therapeutic compound [32]. Promising results have also been obtained when a combination of DOX with parthenolide (sesquiterpene lactone) was tested in cancer cells [33,34].

With the aim of elucidating the mechanism of sesquiterpenes-mediated enhancement of DOX toxicity in cancer cells, two main hypothesis were considered: A further increase of oxidative stress caused by DOX and/or increased intracellular DOX accumulation in cancer cells. It is well known that cancer cells display increased basal oxidative stress and they are vulnerable to agents that further augment ROS levels [35]. For this reason the use of pro-oxidant agents represents a possible strategy to selectively target tumor cells (e.g., [36,37]). As the pro-oxidative effects of many sesquiterpenes have been documented [3], we decided to assay sesquiterpene-mediated ROS formation and the effect of sesquiterpenes on DOX-caused ROS formation.

**Figure 6:**
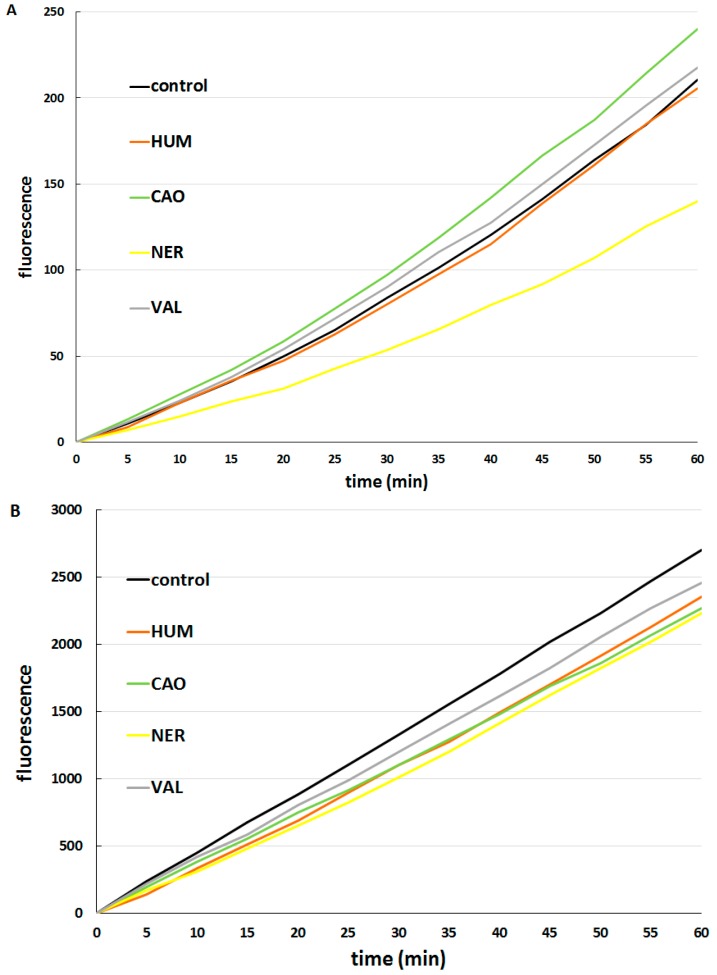
Effect of sesquiterpenes (10 µg/mL) on ROS formation in CaCo-2 cells (**A**); and hepatocytes (**B**).

The effect of individual sesquiterpenes on ROS formation was tested and compared in CaCo-2 cells (see Figure 6A) and hepatocytes (see Figure 6B). In CaCo-2 cells, marked differences among the effects of individual sesquiterpenes were observed. While NER acted as a strong antioxidant, CAO and VAL had pro-oxidative effects. HUM did not influence ROS formation. On the other hand, all sesquiterpenes tested exhibited mild antioxidant properties in hepatocytes.

These results are in accordance with a lack of toxicity of these sesquiterpenes in hepatocytes. Antioxidant activities of NER have previously been reported by Pacifico *et al.* [38], while in another study *cis*-nerolidol was able to scavenge hydroxyl radical (•OH) in radical scavenging assays, but HUM did not show any scavenging effect [39].

**Figure 7:**
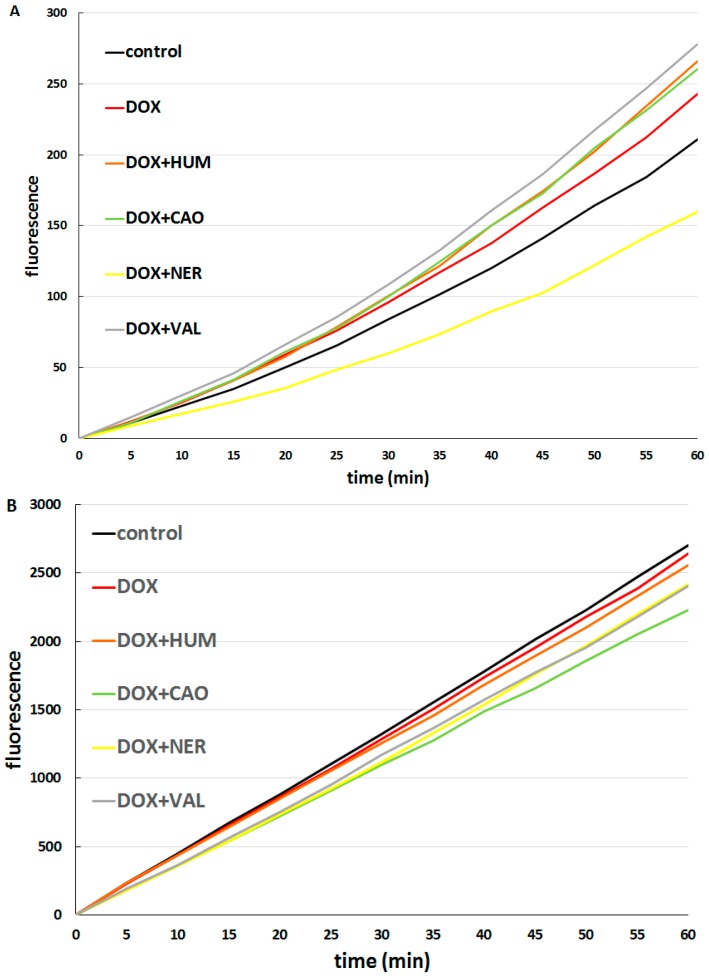
Effect of DOX (1 µM) alone and DOX (1 µM) + sesquiterpene (10 µg/mL) combinations on ROS formation in CaCo-2 cells (**A**); and hepatocytes (**B**).

Consequently, the formation of ROS was assayed in cells treated with DOX alone and in combinations of DOX with each sesquiterpene. DOX alone increased ROS production in CaCo-2 cells but did not have a significant effect in hepatocytes. In CaCo-2 cells (see Figure 7A), the combination of DOX with NER had a significant antioxidant effect, while combinations of DOX with other sesquiterpenes significantly increased ROS formation in comparison to the effect of DOX alone. This finding indicates that the synergism of NER and DOX in antiproliferative activity cannot be based on increased ROS production. In hepatocytes (see Figure 7B), all combinations of DOX with sesquiterpenes decreased ROS formation as compared with DOX alone.

**Figure 8:**
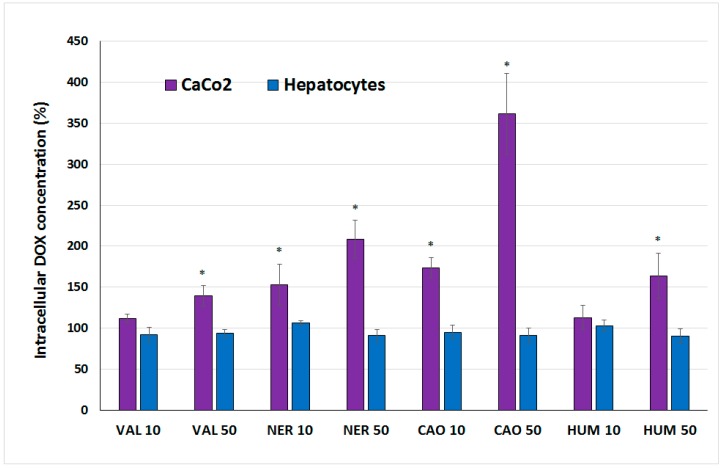
Effect of sesquiterpenes (10 and 50 µg/mL) on DOX intracellular concentration in CaCo-2 cells and hepatocytes. Data (mean ± S.D. from triplicates) are expressed as percentage of DOX intracellular concentration in cells treated with DOX alone (=100%). The asterisk indicates a significant difference from the control cells (*p* < 0.05).

The effect of sesquiterpenes on DOX accumulation in cells was also studied. Concentrations of DOX within cells after a 3-h incubation of CaCo-2 cells, hepatocytes with DOX alone and DOX + sesquiterpene combinations were analyzed. The obtained results (presented in Figure 8) showed marked differences between each type of cells. In hepatocytes, the addition of sesquiterpenes did not affect intracellular DOX concentration. On the other hand, the addition of all sesquiterpenes to DOX led to a concentration-dependent increase of DOX concentration within CaCo-2 cells. NER and CAO caused a more pronounced enhancement of DOX accumulation than HUM and VAL. CAO (at concentration 50 µg/mL) was able to enhance the intracellular DOX concentration in CaCo-2 cells at a rate of 3.6-times.

The marked ability of CAO to selectively increase DOX concentration within cancer cells could explain the strong synergism of CAO with DOX in CaCo-2 cell killing despite the relatively low antiproliferative effect of CAO alone. Concurrently, CAO was also able to increase DOX-mediated ROS production and thus both mechanisms likely contribute to CAO and DOX synergism in cancer cells. However, other actions may be also involved, as several various mechanisms in CAO anticancer activities have been studied, with many interesting results obtained [8,25,27]. For all these reasons CAO can be considered a promising compound for further testing regarding its possible use in combination anticancer therapy.

## 3. Experimental Section

### 3.1. Chemicals and Reagents

Eagle’s minimum essential medium (EMEM), Dulbecco’s modified Eagle medium (DMEM), *N*-2-hydroxyethylpiperazine-*N*′-2-ethanesulfonic acid (HEPES) buffer, MEM non-essential amino acid solution and neutral red were supplied by Sigma-Aldrich (Prague, Czech Republic). Fetal bovine serum and gentamicin sulfate were purchased from Invitrogen (Carlsbad, CA, USA) and bovine serum albumin (BSA) from Fluka (Prague, Czech Republic). Doxorubicin and pure sesquiterpenes (α-humulene, β-caryophyllene, β-caryophyllene oxide, *trans*-nerolidol, valencene) were supplied by Sigma-Aldrich. Stock solutions were prepared in dimethyl sulfoxide (DMSO) and stored in the dark at 4 °C. All other chemicals used were of HPLC or analytical grade and provided by Sigma-Aldrich.

### 3.2. Cancer Cell Culture

The human epithelial colorectal adenocarcinoma line CaCo-2 was purchased from ATCC (supplier for Czech Republic: LGC Standards, Lomianki, Poland). Cells were multiplied in three passages, frozen in aliquots and stored in liquid nitrogen. The absence of mycoplasma in all cell lines used in the laboratory was periodically checked by Generi Biotech (Hradec Kralove, Czech Republic). For every set of experiments (lasting 3–9 weeks) new storage cells were resuscitated. The CaCo-2 cells were cultured in MEM supplemented with 10% heat-inactivated FBS, 1% non-essential amino acid, 1.5% l-glutamine solution and 0.5% penicillin/streptomycin. Cells were grown in T-75 cm^2^ culture flasks in a humidified atmosphere containing 5% CO_2_ at 37 °C.

### 3.3. Preparation of Primary Culture of Rat Hepatocytes

The rat hepatocytes were obtained from rat liver by the two-step collagenase method. Briefly, the liver was perfused with salt solution (0.14 M NaCl, 5.0 mM KCl, 0.8 mM MgSO_4_) in Na^+^/K^+^ phosphate buffer (0.2 mM, pH 7.4) containing a calcium binding component (0.4 mM EGTA). Consequently, the liver was perfused with phosphate buffer containing calcium chloride (1.46 mM) and collagenase (30 mg/100 mL) at 37 °C. The collagenase perfusion was proceeded for 5–6 min. After perfusion the liver was transferred to a medium containing BSA and the hepatocytes were released. The obtained suspension was filtered through a nylon mesh and centrifuged at 40× *g* for 5 min at 4 °C. The pellet was re-suspended in chilled buffer and the washing procedure was twice repeated. Finally, three million viable (75%–85%) cells in 3 mL of culture medium ISOM (1:1 mixture of Ham F12 and Williams’ E) were placed into 96-well plastic dishes. The fetal calf serum was added in the culture medium (5%). Cultures were maintained without the tested compounds for 4 h at 37 °C in a humid atmosphere of air and 5% CO_2_.

### 3.4. Tests of Cell Viability

Pure commercially available sesquiterpenes and DOX were pre-dissolved in DMSO or in pure ethanol. The concentration of organic solvent in medium was 0.1%. The cells were exposed to various concentrations of the tested compounds in culture medium. The cells cultured in medium with pure DMSO or ethanol without tested compounds were used as control samples. DMSO (10%) served as a positive control in both the primary culture of hepatocytes and of CaCo-2 cells. The number of viable CaCo-2 cells was assayed using the neutral red uptake test after 72 h expositions. The viability of the hepatocytes was tested using a MTT assay after 24 h expositions.

#### 3.4.1. The Neutral Red Uptake (NRU) Test

The cells were cultured in 96‑well plates. After 72 h exposure, the medium was removed and 100 µL of neutral red-containing medium was added into each well, at which time the plates were incubated for an additional 3 h at 37 °C. The cells were washed with 100 µL of PBS, then fixed in a solution of 0.5% formaldehyde/1% calcium chloride for 15 min. The neutral red dye was extracted from the viable cells with a solvent (50% ethanol/1% acetic acid) by shaking for 30 min at room temperature. The absorbance of solubilized dye was measured using a spectrophotometer (Infinite M200, Tecan, Männedorf, Switzerland) at 540 nm. Each sample was assayed in 6 parallels and 3 independent experiments were performed. The viabilities of the treated cells were expressed as a percentage of untreated controls (100%). The effect of sesquiterpenes on DOX efficacy was evaluated using CalcuSyn software.

#### 3.4.2. The MTT Assay

The mitochondria of living cells are able to reduce yellow MTT (3-(4,5-dimethylthiazol-2-yl)-2,5-diphenyltetrazolium bromide) to purple formazan. The cells were cultured in 96-well plates. After 24 h exposure, 25 µL of MTT solution (3 mg of MTT in 1 mL of PBS) were added to each well. The plates were incubated for an additional 2 h at 37 °C, at which time the medium was removed and the formed formazan was dissolved in 50 µL of 0.08 M HCl in isopropanol by 30 min shaking. The absorbance in each well was read at 570 and 690 nm using a spectrophotometer (Tecan Infinite M200).

### 3.5. Determination of Intracellular Reactive Oxygen Species (ROS) Formation

To assess ROS generation in CaCo-2 cells or in fibroblasts, a measurement of 2′,7′-dichlorodihydrofluorescein-diacetate (H_2_DCF-DA) oxidation was used. This non-fluorescent reagent diffuses passively through the plasma membrane into the cell, where the acetate groups are cleaved by intracellular esterases. The compound can then be oxidized by ROS formed within the cell (particularly by hydroxyl radicals, •OH) to form fluorescent dichlorofluorescein (DCF), while the fluorescence intensity is proportional to the ROS level [40].

CaCo-2 cells or hepatocytes (seeded in a 96-well plate at a density of 2500 cells/well) were washed by PBS buffer and loaded with 100 µL of the tested substances (individual sesquiterpenes, DOX alone, DOX + sesquiterpene combinations pre-dissolved in DMSO) in PBS buffer. After 12-h incubation, 100 µL of 5 μM H_2_DCF-DA were added. After another incubation of 30-min, fluorescence intensity was measured for 50 min at 37 °C using a microplate reader Tecan Infinite M200 at λ_ex_ = 485 nm and λ_em_ = 525 nm. Cells treated with PBS only with pure DMSO (as a solvent) were used as control samples. The 3% H_2_O_2_ solution was used as a positive control.

### 3.6. Test of DOX Accumulation in Cells

The suspension of CaCo-2 cells or hepatocytes (600 µL; density 5000 cells/100 µL) per well was set to a 12-well plate. After 48 h, 600 µL of solutions in medium of the tested substances were added. The cells were treated with DOX (6 µM) alone, or in combination with individual sesquiterpenes (10 or 50 µg/mL) for 3 h. At the end of the experiment, the medium was removed and cells were washed with 500 µL of PBS. One hundred µL of lysing buffer (25 mM Tris, 150 mM NaCl, 1% Triton, pH 7.4) were then added, at which time the cells were lysed for 15 min, then mixed with 300 µL of ice-cold methanol and centrifuged (10,000× *g*, 10 min, 4 °C). The supernatant was filtered through a 0.22 µm filter and the filtrate was used for analysis.

DOX was detected on an Agilent 1290 Series UHPLC chromatographic system (Agilent, Santa Clara, CA, USA) equipped with a Zorbax C18 Eclipse Plus (2.1 × 50 mm, 1.8 μm) column with a 1290 Infinity inline filter (Agilent). The HPLC method used by [41] was adapted to a UHPLC system: Isocratic elution of 1.0 mL/min by 0.1% formic acid in water and acetonitrile in ratio 76:24; 30 °C thermostated column compartment; fluorescence detection at λ_ex_ = 480 nm and λ_em_ = 560 nm. Chemical standards were purchased from Toronto Research Chemicals (Toronto, ON, Canada).

### 3.7. Statistical Analysis

Data are presented as means ± S.D. of a given number of experiments. Statistical significance (evaluated using a one-way ANOVA) was acceptable to a level of *p* < 0.05. The concentration inducing a 50% decrease of cell viability as compared to control (IC_50_) was calculated using GraphPad Prism 6.0 nonlinear regression.

## 4. Conclusions

Taken together, HUM, NER, VAL and CAO inhibited the proliferation of CaCo-2 cancer cells but did not affect the viability of hepatocytes. CAO, NER and VAL synergistically potentiated the efficacy of DOX in cancer cell killing. When the potential mechanism of the action of sesquiterpenes was studied, all sesquiterpenes exhibited the ability to selectively increase DOX accumulation in cancer cells and did not affect DOX concentration in non-cancerous cells. Additionally, CAO and VAL were able to increase the pro-oxidative effect of DOX in cancer cells. Moreover, CAO mildly ameliorated DOX toxicity in hepatocytes.
